# Notes and News

**Published:** 1905-08-12

**Authors:** 


### Notes and News.

It is suggested that the mail steamers laid up in the Solent as out of date could be used as sanatoria for consumptive children.

During the visit of the French Fleet to Cowes, some of the officers paid a visit to the King's Convalescent Home for Officers at Osborne.

The Public Health Amendment and the Medical Act Amendment Bills, were both read for the third time before the House of Lords on Tuesday and passed.

A widow in Clerkenwell has been awarded £40 against a fishmonger who sold stewed eels to her son, after partaking of which he died. The verdict is an important one, as the Court gave judgment against the fishmonger because he was responsible for the goodness of the food as the customer could have no opportunity of selection. In the case of uncooked food the customer has the power of selection or rejection.

A meeting of the Council of the Metropolitan Hospital Sunday Fund will be held on August 11th, at 4 o'clock, at the Mansion House to recommend an alteration in the laws of the constitution so as to enable the Queen Victoria Jubilee Institute for Nurses to receive a grant and recommend an increase in the secretary's salary, and to order payment of awards to hospitals and dispensaries, and other business. The collection for this year already exceeds £75,000.

Mr. Herman K. Burney has just been appointed secretary of the Royal Berkshire Hospital, Reading. Mr. Burney, who is a B.A. of Trinity College, Cambridge, and a barrister-at-law, has been steward of the *Dreadnought* Hospital, Greenwich, for the last two and a-half years, having joined the staff of that institution with a view of obtaining the experience which would fit him for the post of secretary of an important hospital. In congratulating Mr. Burney on his selection we may point out that he no doubt owes his success, like a number of other hospital secretaries, in a large measure to the valuable training he received at the *Dreadnought* Hospital, Greenwich. We anticipate therefore that Mr. P. J. Michelli, who is president of the Hospital Officers' Association, will be inundated with applications for the post Mr. Burney vacates from a number of gentlemen who are anxious to obtain the necessary training in hospital work to fit them for the higher posts in the hospital world.

The International Congress of Anatomy was opened on August 7th at Geneva. There was a large attendance of representatives of European and American universities. Dr. Eternod of Geneva delivered the opening address and Mr. Symington of Belfast acted as president during the business which followed. The Congress has arranged a meeting at Boston in 1907.

Last week a deputation of members of Parliament, including Dr. Hutchinson and Dr. Thompson, waited upon the President of the Local Government Board, and laid before him a number of facts and figures relating to infant mortality in certain districts. Mr. Gerald Balfour, in reply, said that he would have inquiries made by special officers of his department in the various cities where the rate of infant mortality was abnornal, and that he would carefully consider their report with the view to taking action to remedy the evil.

By the sudden death on Tuesday of Mr. Christopher Heath the surgical profession has lost one of its most prominent figure-heads. Becoming a member of the Royal College of Surgeons in 1856, Mr. Heath was elected a Fellow of the College four years afterwards. In 1862 he was appointed assistant surgeon to the Westminster Hospital, a post which he relinquished a few years later on becoming a member of the surgical staff of University College Hospital. In addition to being one of the ablest operators, Mr. Heath had a world-wide reputation as a clinical teacher and author. His manual of "Minor Surgery" passed through no fewer than 12 editions, and his "Guide to Surgical Diagnosis" and "Practical Anatomy," among other works, were household names in every medical school. In 1875 Mr. Heath succeeded Sir John Erichsen as Holme Professor of Clinical Surgery at University College, and in 1895 he was elected president of the Royal College of Surgeons.

In his evidence on Friday before the Royal Commission on the Care and Control of the Feeble-minded, Dr. Bedford Pierce, medical superintendent of the Retreat at York, said that it was, perhaps, impossible to do anything to control the liberty of adults who had already been at large in the world unless they were decidedly insane or had become criminals; but he submitted that those children who had exhibited marked mental deficiency at school should not be allowed to go freely into the world until there was reason to believe that the mental deficiency had disappeared. Assuming that special schools were available for all children until they were 16 years of age, he would support the suggestion that special industrial colonies be established for children above that age, and in these every effort should be made to discover their special faculties, and if possible to teach them how to earn a living. It was only reasonable that children who had required special and costly education should be apprenticed to the State until they were 20 years of age, and that their future should depend upon their success while serving their time. If the feeble-minded on leaving school were practically prevented from marrying by segregation, there would be a considerable reduction in the number of insane, epileptic, and defective persons.

